# Monolayer and Bilayer Formation of Molecular 2D Networks Assembled at the Liquid/Solid Interfaces by Solution-Based Drop-Cast Method

**DOI:** 10.3390/molecules26247707

**Published:** 2021-12-20

**Authors:** Xingming Zeng, Yi Hu, Rongbin Xie, Sadaf Bashir Khan, Shern-Long Lee

**Affiliations:** 1 Institute for Advanced Study, Shenzhen University, Shenzhen 518060, China; zengxingming@email.szu.edu.cn (X.Z.); huyiyangyang@126.com (Y.H.); xierongbin22@163.com (R.X.); sadafbashirkhan@yahoo.com (S.B.K.); 2 Key Laboratory of Optoelectronic Devices and Systems of Ministry of Education and Guangdong Province, College of Optoelectronic Engineering, Shenzhen University, Shenzhen 518060, China

**Keywords:** TPTC, bilayer, molecular self-assembly, STM, HOPG

## Abstract

In recent years, extending self-assembled structures from two-dimensions (2D) to three-dimensions (3D) has been a paradigm in surface supramolecular chemistry and contemporary nanotechnology. Using organic molecules of p-terphenyl-3,5,3′,5′-tetracarboxylic acid (TPTC), and scanning tunneling microscopy (STM), we present a simple route, that is the control of the solute solubility in a sample solution, to achieve the vertical growth of supramolecular self-assemblies, which would otherwise form monolayers at the organic solvent/graphite interface. Presumably, the bilayer formations were based on π-conjugated overlapped molecular dimers that worked as nuclei to induce the yielding of the second layer. We also tested other molecules, including trimesic acid (TMA) and 1,3,5-tris(4-carboxyphenyl)-benzene (BTB), as well as the further application of our methodology, demonstrating the facile preparation of layered assemblies.

## 1. Introduction

Surface supramolecular self-assembly provides a simple method for generating nanostructured organizations on a solid surface at nanoscale precision [1,2,3,4,5]. Understanding the orientation control of the molecular building blocks in supramolecular assemblies is an important aspect for achieving high performance in organic photovoltaics (OPVs), field-effect transistors (OFETs), or light-emitting diodes (OLEDs) [6]. In the field of organic electronics, electron transfer through π–π stacking is the main idea in designing various compounds, which has led to the emerging field of discotic liquid crystals (DLCs) [7]. Typically, the face-on and edge-on configurations are desired in the OPV and OFET devices, respectively [8]. The conformations in molecular self-assemblies are generally studied using scanning tunneling microscopy (STM) with sub-molecular resolution [9]. The substrate can be graphite, Au (111), Ag (111), Cu (110), or others because of their intrinsic atomic flat surface [10,11,12,13,14,15]. In general, strong interactions between molecule and substrate occur in the case of large aromatics in DLCs [16]. Considering electron transport, much effort has been devoted to methodological developments toward molecular alignment for tilting DLCs into horizontal π–π stacking on a solid surface. Methods involve Langmuir–Blodgett technique, zone casting, shear flow, and electric/magnetic fields, as well as the utilization of alignment layers, to name just a few [17,18].

The DLC phases have been widely characterized by STM, scanning electron microscopy (SEM), transmission electron microscopy (TEM), atomic force microscope (AFM), and two-dimensional wide-angle X-ray scattering (2D-WAXS) [19,20,21]. Exploration works of either monolayers or multilayers have been reported [22,23,24,25,26]. On the other hand, at the liquid–solid interface, molecular patterning is mainly based on the principle of self-assembled monolayers (SAMs). In this field, so far the exploration of multilayers has been rarely reported compared with the appreciable literature related to monolayers [27,28,29,30,31]. This may result from the greater level of complication in the mechanism, involving molecule–molecule and molecule–substrate attractions, especially the interactions occurring along the vertical direction to the substrate surface.

Despite the significance, to date, it has been scarcely investigated regarding the methodological advances for controlling supramolecular stacking along the vertical direction to the substrate. It requires to fine-tune the sophisticated balance for a given system. To address such a challenge, that is, extending supramolecular self-assembly toward vertical growth, here we report a simple yet effective method for tailoring vertical π–π stacking of a series of π-conjugated molecular systems. Based on these systems, we have previously systematically explored the monolayers induced by factors, such as the bias and host-guest co-assemblies, and the dynamic of vertical growth [32,33,34,35,36]. We demonstrate our approach by firstly testing the system of *p*-terphenyl-3,5,3′,5′-tetracarboxylic acid (TPTC) at the octanoic acid (OA)/highly oriented pyrolytic graphite (HOPG) interface. The stable and large-area bilayer networks were successfully formed. To demonstrate the usefulness of our method, we also investigated other molecular systems including trimesic acid (TMA) and 1,3,5-tris(4-carboxyphenyl)-benzene (BTB).

## 2. Experimental

All the STM experiments were conducted after a drop of sample solution was deposited onto a HOPG (quality ZYB, Bruker, USA) surface at ambient condition (Temperature: 22–24 °C; Humidity: 45–50%), in constant current mode, using the Keysight 5500 system. Solvents: OA (≥99%) and phenyl octane (PO, ≥98%), target molecules: TPTC (≥95%), TMA (≥98%), and BTB (≥95%) were commercially purchased and used without further purification. The STM tips were mechanically cut from Pt/Ir wires (80/20, diameter: 0.25 mm). TPTC samples dissolved in OA were prepared from two types of solutions: one was sonicated for 1 h and the other was just shaken by hand for 3 s. Upon being mixed (volume ratio = 1:1) and deposited onto the HOPG surface, the sample was monitored by STM. The scanning parameters were described in the figure captions. The STM images were captured line-by-line via the STM tip. The scanning speed of STM is constant, and was set as 0.602 μm/s in this case. The time interval between two successive images was 129 s. The STM images were analyzed by either WSxM or SPIP software (scanning probe image processor, Image Metrology ApS). The proposed structural models were built based on the high-resolution STM images, using the Materials Studio 7.0 software.

## 3. Results and Discussion

### 3.1. Methodology

**Scheme 1:**
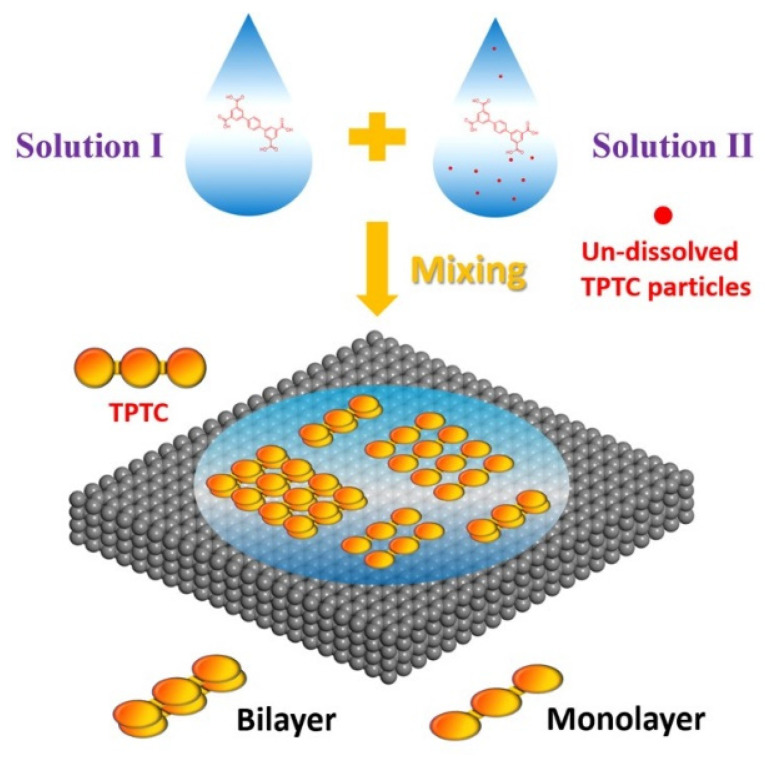
The schematic illustration representing the method developed in this work for forcing the vertical growth of TPTC. Solution I in which all the TPTCs were dissolved was prepared by one-hour sonication. Solution II in which some un-dissolved TPTCs existed was shaken by hand for 3 s.

In our approach, the procedure involves the mixing of two kinds of solutions (for each solution 3 mL, Scheme 1). *Solution I* is the saturated one, in which TPTCs were dissolved in OA and sonicated for 1 h. *Solution II* was prepared by placing some TPTC powders (ca. 1 mg) into the pure OA solution (2 mL) which then was shaken by hand for 3 s, resulting in naked-eye-visible TPTCs (un-dissolved particles) in the solution. Then, *solution I* and *II* were mixed, and a droplet (~20 μL) of this mixture was deposited onto the HOPG surface for preparing the sample. Time is an important factor for the formation of bilayer structure. *Solution II* must be used within 30 min upon being prepared, or it does not work on forming bilayer, because the un-dissolved TPTC powder will gradually dissolve into the solvent over time. In our methodology, *solution I*—in which TPTCs were well-dissolved—makes sure that the formation of the monolayer (the bottom layer) normally proceeds. The function of *solution II*—in which there are enough un-dissolved TPTCs—is to provide the molecule source for forming the bilayer (top layer). These two solutions functioning at the same time is the essential condition that guarantees the bilayer.

### 3.2. Bilayer Formation via TPTC Self-Assembly

**Figure 1:**
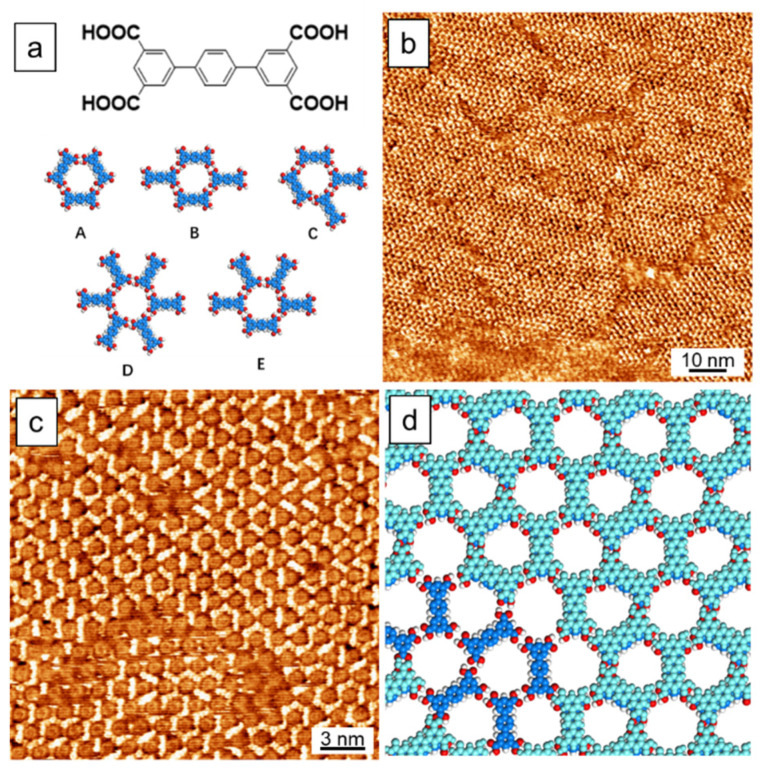
(**a**) Chemical structure of TPTC and five possible arrangements constituting the glass-like random-tiling assembly. (**b**,**c**) Large-scale and high-resolution STM images for the TPTC self-assembly at the OA/HOPG interface. Scanning parameters: *V_s_* = −0.9 V, *I_t_* = 75 pA for (**b**) and 100 pA for (**c**). (**d**) A proposed bilayer model where molecules in the bottom and top layers are in dark and light blue, respectively.

TPTC consists of three connected benzene rings and four carboxyl groups which thus can form hydrogen bonds during the self-assembly process. In the self-assembled monolayers, one can observe five kinds of pores built by different arrangements of TPTC, labeled as A, B, C, D, and E (Figure 1a). These pores were previously reported by Beton et al. for their initial investigation of TPTC [37]. Recently, De Feyter et al. also investigated different polymorphs of TPTC by initiating nucleation and growth [38]. They focus on the impurity-induced phase transition and growth towards the third dimension, which is followed by molecular removal via the STM tip. The five kinds of pores appear randomly in the molecular layers, and hydrogen bonding, π–π stacking, and van der Waals interaction are expected to play important roles in stabilizing the glass-like networks. Note that the bilayer formation of TPTC was also studied by Beton et al. [39]. They found that fullerene (C_60_), due to its intrinsic 3D structure, can assist the formation of the top layer of TPTC, leading to 3D hybrid architectures. Figure 1b,c show the large and small scale STM images of the bilayers of TPTC prepared based on our “guest-molecule-free” method (Scheme 1). The bottom layer displays a dark contrast, covering almost the entire HOPG surface. In our procedure, the sonication treatment for *solution I* is to promote the monolayer formation, whereas the low solubility of *solution II* is to force the occurrence of the vertical stacking of TPTCs on the surface. The domains that show light contrast represent the top layer of TPTC. For ease of distinguishing the height difference between the monolayer and bilayer structures, the image of line profile which crosses the monolayer and bilayer domains are shown in Figure S1 (see Supplementary Material). The contrast reversing issue in STM images for the assemblies were discussed in Figure S2 (see Supplementary Material) in the supporting information. Figure 1d is the proposed structural model for the double layer assembled networks, with a small area of monolayers. The top and bottom layers are in different colors.

**Figure 2:**
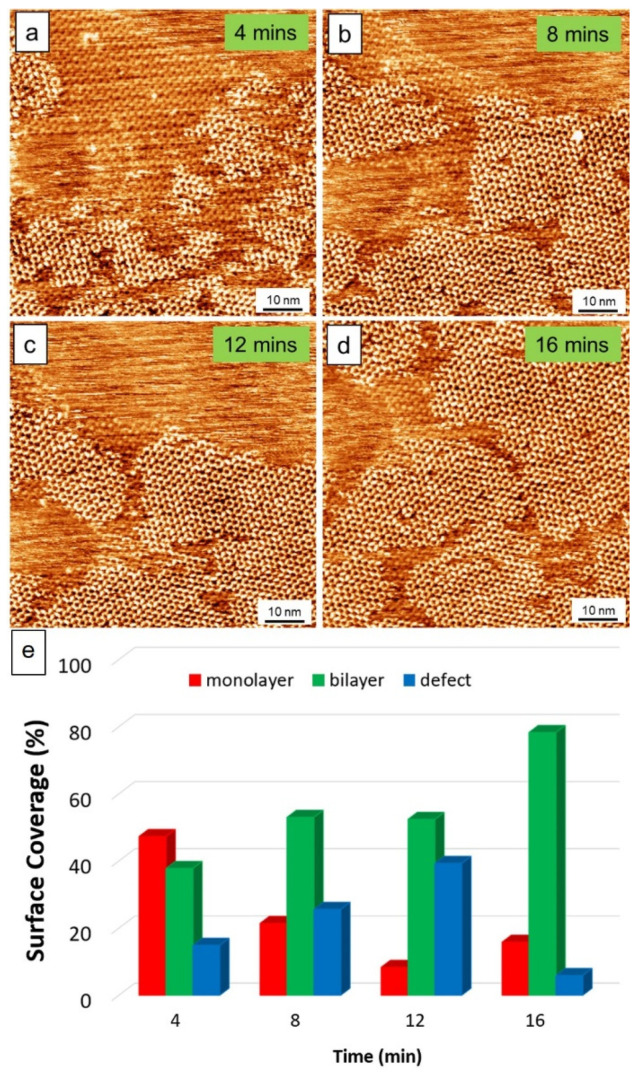
(**a**–**d**) STM images showing the dynamics of the formation of bilayer TPTC. Scanning parameters: *V_s_* = −0.9 V, *I_t_* = 75 pA. (**e**) Statistics of the surface coverage for the monolayers, bilayers, and defects at different time. As the scanning time prolongs, some molecules can adsorb onto the surface of the monolayers. Thus, the monolayers gradually transfer into bilayers.

Figure 2 presents the STM monitoring of the dynamics of forming the layered structure of TPTC at the OA/HOPG interface. The consecutive imaging was started upon the STM tip reaching the surface, which was about 5 min after depositing the sample solutions onto the surface. The tendency toward yielding bilayers was revealed by STM. In the beginning, the formation of the monolayers occurred fastest but the formation of bilayers was prompt. The growth dynamic of the top layer was captured frequently. About 16 min later, around 80% of the scanning area (80 × 80 nm^2^) had been covered by the bilayers (Figure 2d, Table S1, Supplementary Material). In general, one hour later, around 90% of the scanning area was covered by the bilayer. Figure 2e shows the analysis of the phase transformation according to the statistics from Figure 2a–d. Figure S3 and Table S2 (see Supplementary Material) exhibit another large-area STM imaging of the dynamics, which shows a tendency consistent with that for the growth of bilayer in Figure 2e. The defect means area without molecules (Figure S4, Supplementary Material). An ex-situ experiment carried out led to the same result, indicating that the formation of the bilayers is less associated with STM bias.

The as-prepared layered structures are quite stable compared with the monolayers formed spontaneously. For TPTC, we recently reported that its structural polymorphism in self-assembled networks can be easily induced via switching STM bias (substrate polarity) [40]. A positive STM bias can readily induce the closely packed assemblies generating the glass-like random-tiling structure. In contrast, Figure S5 (see Supplementary Material) shows an exercise where the bilayers of TPTC were less sensitive after the STM polarity was changed, indicative of their extremely high stability. We reasoned that the stability of the bilayers was enhanced by the horizontal hydrogen bonding interactions of the top layer and the vertical π–π stacking between the layers in the 3D organization.

During our STM explorations, we have not obtained three or more films. This is consistent with the simulation run by Artur Ciesielski et al. [41], suggesting that part of the TPTC molecule at the upper layer is tiled. As the X-layer (X > 2) appears, the layer–substrate van der Waals interaction becomes weaker. Therefore, the X-layer (X > 2) is not thermodynamically stable enough to exist at the liquid/solid interface.

### 3.3. Reversibility between the Formation and Removal of the Second Layer

Previously in the literature it was reported that the bilayer formation only occurs with the assistance of C_60_ molecules, and the removal of the top layer can only take place in the presence of coronene molecules [39]. We have reported how to generate the bilayers. Figure S6 (see Supplementary Material) presents the method for reversing the bilayers to monolayers. The mechanical force from an STM tip “contact/disturbance” was found able to remove the upper layers. The process was to increase the tunneling current from 75 to 300 pA, resulting in the reduced distance between the STM tip and substrate underneath. The self-assembly film forms on the substrate, according to the three distinct stages in crystallization: nucleation, free growth, and ripening [40]. The second film forms on the monolayer, starting from the sites with structural defects, or at the domain boundaries. Contrarily, the removal of the second layer occurs in the reverse, meaning that the areas near structural defects and domain boundaries are the last to be removed to form back to monolayer.

### 3.4. Application of the Methodology for Other Molecule Systems

**Figure 3:**
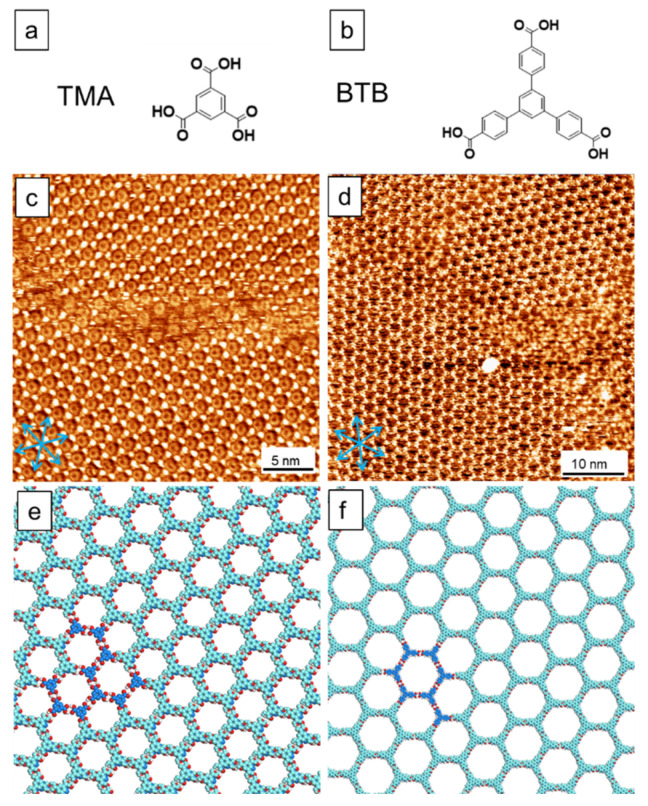
STM images show the bilayer formation via our methodology. (**a**,**b**) Chemical structures for TMA and BTB. (**c**,**d**) Self-assembled bilayer structures for TMA and BTB. (**e**,**f**) The corresponding models. The blue arrows in the STM images indicate the HOPG direction. The dark contrast in STM images reveals the monolayers, namely, the bottom layer of either TMA or BTB system. The dark and light blue parts in the models indicate the bottom and top layers of the packing. Scanning parameters: *V_s_* = −0.9 V, *I_t_* = 75 pA.

Compared to the literature, [39] our methodology represents a straightforward method for efficiently manipulating vertical supramolecular self-assembly. Figure 3 presents the bilayers of other molecular systems forced to form via the unusual control, including the well-studied systems of TMA and BTB (Figure 3a,b), demonstrating the universality of the herein reported approach. In Figure 3c,d, the majority of the STM images are covered by the bilayer phases, which can be distinguished from the monolayer phases by the contrast difference. The images of the line profile across the monolayer and bilayer domains are shown in Figure S7 (see Supplementary Material).

### 3.5. Mechanism

In our methodology *solution I* contains well-dissolved target molecules to ensure the monolayer formation or to maximize the coverage percentage of the first layer over the whole graphite surface. Therefore, one-hour sonication for sample preparation of target molecules was suggested. This can improve the solubility of compounds. The *solution II* contains some powder, which is crucial for layered structure formation. In the present work, we mainly explore aromatic small molecules functionalized with carboxylic acid groups, including TPTC, TMA, and BTB at the organic solvent/graphite interface. Below we discuss some aspects of monolayer, bilayer, and multilayer formations including molecular structure, packing fashion, dynamics, sample concentration, sonication, impurity, polymorphs, solvent role, and the mass transfer principle. 

#### 3.5.1. Monolayers, Bilayer, and Multilayers

Supramolecular self-assembled monolayers formed at the liquid–solid interface can be easily obtained and elucidated by thermodynamics. An elegant example is the self-assembled alkyl thiol monolayer on Au (111) [42]. For the present studied systems, past research with STM has obtained the knowledge that the synergic interactions of molecule–molecule hydrogen bonds and molecule–graphite π attractions are the main driving forces to stabilize the molecular monolayers physisorbed on the surface. Such forces can maintain low-density porous assemblies, although they are relatively unstable compared with closely packed ones. In the case of bilayer scenario, one can envisage that horizontal and vertical forces need to be involved simultaneously to afford the stabilization of the layered organization. The same conditions including intermolecular hydrogen bonds are required to horizontally maintain the two sheets of molecular networks and, moreover, π–π attraction between the two layers are additionally needed. Under a ultra-high vacuum (UHV) condition, the layered π-stacking of aromatic small molecules can be readily prepared by molecular evaporation. It seems it will be simple to express the mechanism, however, it is quite complex when coming to the liquid–solid interface environment. The difficulty of the preparation of multilayer structures arises due to the well understood, but difficult to control dynamics of the molecular behavior at the interface, including adsorption, desorption, and lateral diffusion, as well as the dynamic equilibrium. Sometimes, depending on the given molecular systems, polymorphic assemblies referring to more than one type of packing can occur. The yielding of the stacking layer also depends on various conditions and molecular systems. For example, polycyclic aromatic hydrocarbons (PAHs) may facilitate the formation of vertical columnar packing due to strong π–π stacking between PAHs. For the aromatic small molecules discussed here (TPTC, TMA, and BTB), molecular desorption from the graphite surface into the liquid phase tends to occur. Some parts of the surface that remain empty can thus be ascribed to dynamic solvent adsorption and desorption. 

#### 3.5.2. Sample Concentration, Sonication, and Impurity

Spontaneously, a high-concentration sample solution may promote the occurrence of layered structure formations. Sonication treatments of sample solutions can improve the solubility and thus increase solute concentrations. However, based on our investigations, long-time sonication of sample solutions also resulted in monolayers for the case of TMA although, for the TPTC case, we observed the presence of bilayers occasionally. Recently, De Feyter et al. reported the unexpected finding of TPTC bilayers, ascribed to the existence of the impurities of commercially available compounds (Sigma-Aldrich, 99.9%) [38]. The result is unexpected because the impurities are ~2.5% based on NMR data. The impurities, so-called side products in organic synthesis, are *p*-quaterphenyl (QPTC) and *p*-quinquephenyl (QQPTC). Molecular modelling (MM) in their work indicated that the adsorption energy of QPTC on graphite (−63.7 kcal/mol) is higher than that of TPTC (−52.0 kcal/mol). The strong ability of π–π stacking of QPTC has thus been proposed to induce the occurrence of the formation of TPTC bilayers. Nevertheless, the impurity-induced bilayer formation cannot be adapted to explain well the case of either TMA or BTB. 

#### 3.5.3. Polymorphs

For the TMA system, Thi Ngoc Ha et al. have reported that long-time sonication of a sample solution can lead to TMA monolayers with increased density [43]. The density of TMA varies as a function of sonication time and overall 5 polymorphic phases have been found. In the literature, the experimental conditions of up to 7 h of sonication were investigated. It is worthwhile to note that no bilayers were observed. On the other hand, for the BTB system, high concentration sample solutions can result in high-density polymorphic phases, including linear and close packing monolayers [44]. Therefore, how to generate layered supramolecular assemblies on a solid surface constitutes a challenging task. Hence, it seems unlikely that high-concentration sample solutions can directly induce the appearance of the second layer especially for porous chicken-wire bilayers. 

#### 3.5.4. Solvation and Solid-to-Solid Mass Transfer

**Scheme 2:**
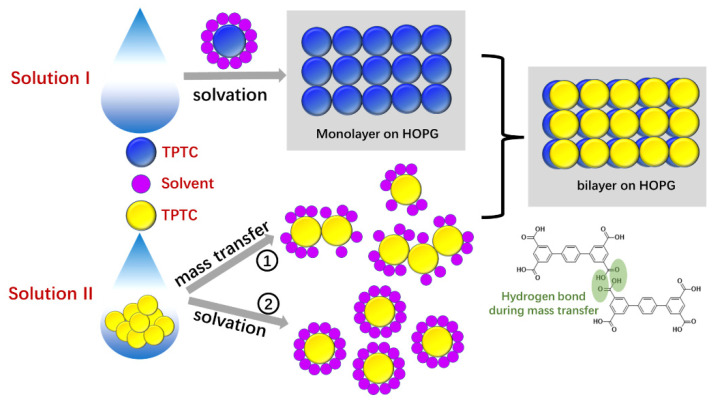
Illustration shows the way of the bilayer formation. For ease of distinguishing, the top and bottom layers of TPTC are in yellow and blue circle shapes, respectively. The purple circle represents the solvent molecule.

Given that formation of porous bilayer structures is counterintuitive, we now report a novel methodology involving the use of mixed two solutions. We have explained that in our methodology, the *solution I* needs to contain well-dissolved target molecules so as to ensure the monolayer formation. The bilayer formation is expected to be directly related to the *solution II* that needs to contain some powder of the compound. We deposited the two mixed solutions on the graphite surface upon the mixture of the two solutions. The powder in the mixed solution was expected to dissolve gradually. The rate of monolayer formations from the *solution I* is expected to be faster than that of the powder being dissolved because the solvation rate is expected to be low for the *solution II* (no sonication treatment). As presented in Figure 2, our STM has revealed the dynamics in which the monolayers of TPTC have already formed on the surface and the second layer appeared gradually on top of the monolayers. During the dissolving process, the *solution II* is expected to offer a steady source of target molecules including isolated molecules, dimers, trimers, tetramers, and clusters. Note that two possible paths can take place for bilayer formations: (i) The molecules of the powder may dissolve into the liquid phase and later adsorb onto the surface, forming the second layer. However, the solvation of molecules—that is, target molecules encircled by solvent molecules—may influence the ability of molecular self-assembly and thus obstruct the second layer formations. (ii) An alternative path is that the powder in *solution II* may diffuse laterally on the surface and/or over the monolayers, yielding the second layer. This involves the processes whereby the molecules first constitute some parts of the second layer and are followed by self-assembling, based on the nucleation and crystal growth theory. A principle of solid-to-solid mass transfer can explain this phenomenon [21]. Scheme 2 depicts the details of the theory and possible processes.

It is believed that long-time sonication for targeted molecules can lead to good solvation, high solubility, but less aggregation in solution. The aggregated states from *solution II,* including molecules connected by intermolecular hydrogen bonds, might play an important role in the bilayer formation as they could serve as nuclei for further crystal growths of supramolecular assemblies. Moreover, such nuclei can include hydrogen bonds-based dimers and trimers which have a better ability to constitute the second layer compared with isolated target molecules (solvation state of molecules after sonication). This explains why merely using *solution I* does not give rise to bilayer formations. In addition, we also performed further investigations and found that directly placing some powder into the *solution I* that has been sonicated also led to the same results with the mixture solution procedure. This method meets the essential conditions of *solution I* and *II* discussed above for bilayer formations. Nevertheless, the deposition of *solution I* followed by *solution II* later, can produce the full-coverage monolayers and in turn induce the occurrence of bilayer formations on the surface. A series of control experiments of TPTC were run to examine our method (Figure S8, Supplementary Material): (i) Merely using *solution I*, only monolayers exist over the surface (Figure S8a); (ii) Merely using *solution II,* self-assembly seems unlikely to take place as only bare HOPG was revealed under such conditions. (Figure S8b); (iii) Mixing low concentration *solution I* (diluted ten-fold) and *solution II*, bilayers were also observed, though their domains are relatively small (Figure S8c,d); (iv) Sonicating *solution II* until all TPTCs are dissolved, and later mixing it with *solution I*, only monolayers were observed (Figure S8e,f), implying the importance of the powder in *solution II*.

Finally, we also take into account the solvent evaporation effect. We note that the evaporation volume of solvent used is around 1/3 during STM operations (per hours in general). Due to the apparent amount of solvent lost, we cannot rule out the possibility that solvent evaporation may lead to the occurrence of molecular aggregation and thus induce the occurrence of bilayer formations. However, evaporation processes are difficult to control, and in some cases, high-density polymorphic packing yields. Figure S9 (see Supplementary Material) shows the case of BTB where linear packing was transitioned from a porous network after solvent evaporation. This result suggests that the appearance of bilayers is unlikely to result from solvent evaporation although we cannot ignore its contributions during thin-film formations. Remarkably, our case is somewhat like the super-saturation condition, defined as a sample solution state, in which molecules tend to precipitate after an obvious change of environmental parameters such as initially high temperature or high pressure. However, this happens mainly upon environmental changes. Significantly, in our methodology, there is no need to involve the complex control of environmental parameters; the modulation of molecular solubility is easy to perform (as it involves only one type of solvent). Using other types of solvent, such as 1-PO for the TPTC system, we came to the same conclusion (Figure S10, Supplementary Material). This investigation renders our approach simple, general, and straightforward. We thus have described a modest method for preparing supramolecular bilayers. We have showed that the bilayer is stable under the change of electric-field direction of STM (Figure S5). Finally, in Figure S11 (see Supplementary Material) we have presented the UV-Vis data. Compared with *Solution I*, *Solution II* displayed a slight red shift for their peaks. Moreover, the peaks of the mixture correspond well to *Solution I* and *II*. We ascribe this slight peak shift to the π-stacking interaction in different molecular aggregations, which is a commonly existing force among the aggregated π-conjugated molecules. This UV-Vis data can further support our proposal of the mechanism (less influence from solvation, Scheme 2). In a nutshell, rather than requiring multiple deposition steps, the bilayer assembly based on our simple drop-cast method was driven by the basic self-assembled principle, and, once initiated, the self-assembly can progress without additional external force or stimuli.

## 4. Conclusions

In conclusion, we have reported a simple solution-processing method for bottom-up yielding of layered molecular assemblies, which cannot form on surfaces spontaneously. As has been commonly known, supramolecular self-assembly usually happens at the liquid–solid interface within 2D, [45,46] while crystal growth towards the third dimension seems to be hard. Our method constitutes a protocol that involves the mixture of two types of solutions that have different solubility, by which we have shown via STM that upon deposition, the vertical growth of TPTC on the bottom layer of self-assembled networks can take place gradually. The as-prepared thin films can be proved to be stable. This methodology is useful and effective for different molecular systems that include TPTC, TMA, and BTB, demonstrating the scope of our strategy. Overall, this work represents a methodological development for precisely growing layered nanostructures that is important both from academic viewpoints and for practical applications. For possible applications, the layered nanostructures would be applicable for functionalizing substrates (e.g., graphene) and hosting guest molecules (e.g., C_60_). Related research is in progress.

## Data Availability

Not applicable.

## Supplementary Materials

The following are available online, Figure S1: Image for TPTC and the line profile which across the monolayer and bilayer domains. Figure S2: Contrast reversing in STM images for the monolayers of TPTC. Table S1: The analysis of the phase transformation between monolayers and bilayers at the OA-HOPG interface. Figure S3: Large-area STM images showing the dynamics of forming bilayers. Table S2: The analysis of the phase transformation between monolayers and bilayers at the OA-HOPG interface. Figure S4: STM images showing the dynamics of the formation of bilayer TPTC. Figure S5: STM images showing the stability of bilayers under electric stimuli. Figure S6: Reserving TPTC bilayers to monolayers via mechanical force. Figure S7: STM image for TMA, BTB and the line profiles which across the monolayer and bilayer domains. Figure S8: STM images for the self-assembly of TPTC in solution I, solution II, 10% diluted solution I and solution II, solution I and fully sonicated solution II. Figure S9: A surface captured 7 hours later after depositing sample I of BTB. Figure S10: Solvent effect of the present system. Figure S11: Spectroscopic analysis of sample solutions.
