# Supplementary material for: Monolayer and Bilayer Formation of Molecular 2D Networks Assembled at the Liquid/Solid Interfaces by Solution-Based Drop-Cast Method
Source: Molecules. 2021 Dec 20;26(24):7707. doi: 10.3390/molecules26247707 (PMC8706512; doi:10.3390/molecules26247707)
Supplement: Supplementary file 1 [file molecules-26-07707-s001.zip › molecules-1492099-supplementary.pdf]

ESI

Monolayer and bilayer formation of molecular 2D networks assembled at the liquid/solid interfaces by solution-based drop-cast method

Xingming Zeng,[1] Yi Hu,[1] Rongbin Xie,[1] Sadaf Bashir Khan,[1,2] and Shern-Long Lee*[1]

[1] Institute for Advanced Study, Shenzhen University, Shenzhen, Guangdong, China 518060

[2] Key Laboratory of Optoelectronic Devices and Systems of Ministry of Education and Guangdong Provence, College of Optoelectronic Engineering, Shenzhen University, Shenzhen, Guangdong, China 518060

Corresponding Author E-mail: sllee@szu.edu.cn (S.-L. Lee)

Content of Supporting Information

Figure S1 Image for TPTC and the line profile which across the monolayer and bilayer domains.

Figure S2 Contrast reversing in STM images for the monolayers of TPTC.

Table S1 The analysis of the phase transformation between monolayers and bilayers at the OA-HOPG interface.

Figure S3 Large-area STM images showing the dynamics of forming bilayers.

Table S2 The analysis of the phase transformation between monolayers and bilayers at the OA-HOPG interface.

Figure S4 STM images showing the dynamics of the formation of bilayer TPTC.

Figure S5 STM images showing the stability of bilayers under electric stimuli.

Figure S6 Reserving TPTC bilayers to monolayers via mechanical force.

Figure S7 STM image for TMA, BTB and the line profiles which across the monolayer and bilayer domains.

Figure S8 STM images for the self-assembly of TPTC in solution I, solution II, 10% diluted solution I and solution II, solution I and fully sonicated solution II.

Figure S9 A surface captured 7 hours later after depositing sample I of BTB.

Figure S10 Solvent effect of the present system.

Figure S11 Spectroscopic analysis of sample solutions.

S1. Experimental data

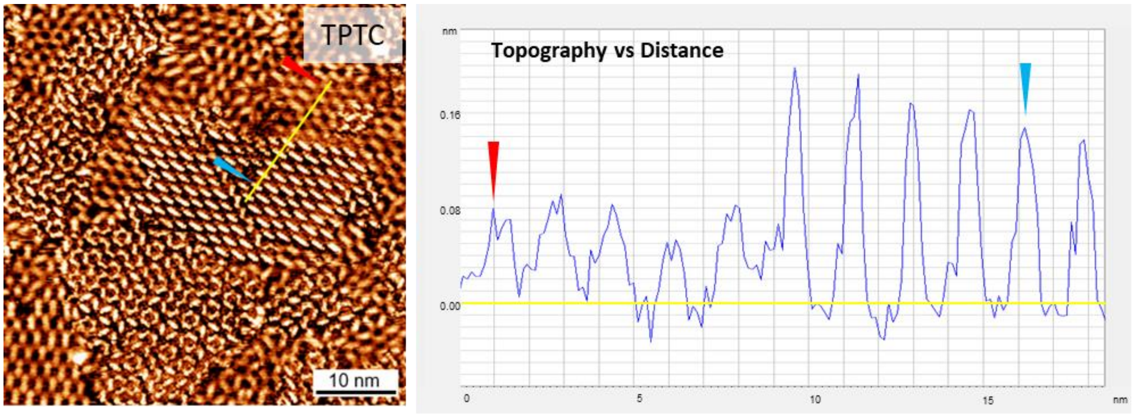

Figure S1. Image for TPTC and the line profile which across the monolayer and bilayer domains. Scanning parameters: V_s = –0.9 V, I_t = 75 pA.

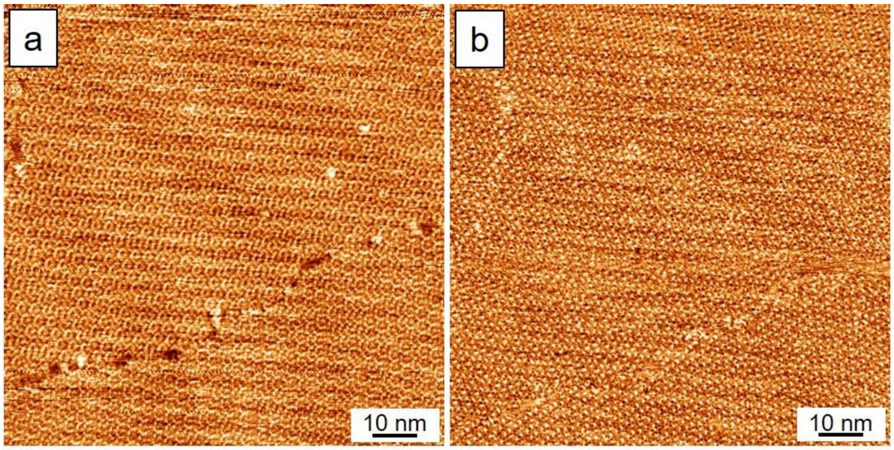

Figure S2. Contrast reversing in STM images for the monolayers of TPTC. Two types of STM images were found for the monolayers of TPTC as reported in the literature. *Vs* = –0.6 V, *It* = 100 pA.

Table S1. Surface coverage for the domains of monolayer, bilayer, and defect, corresponding to the STM images in Figure 2.

Time	Monolayer (%)	Bilayer (%)	Defect (%)
4 min	47.3	37.8	14.9
8 min	21.4	53.0	25.6
12 min	8.3	52.4	39.3
16 min	15.8	78.3	5.9

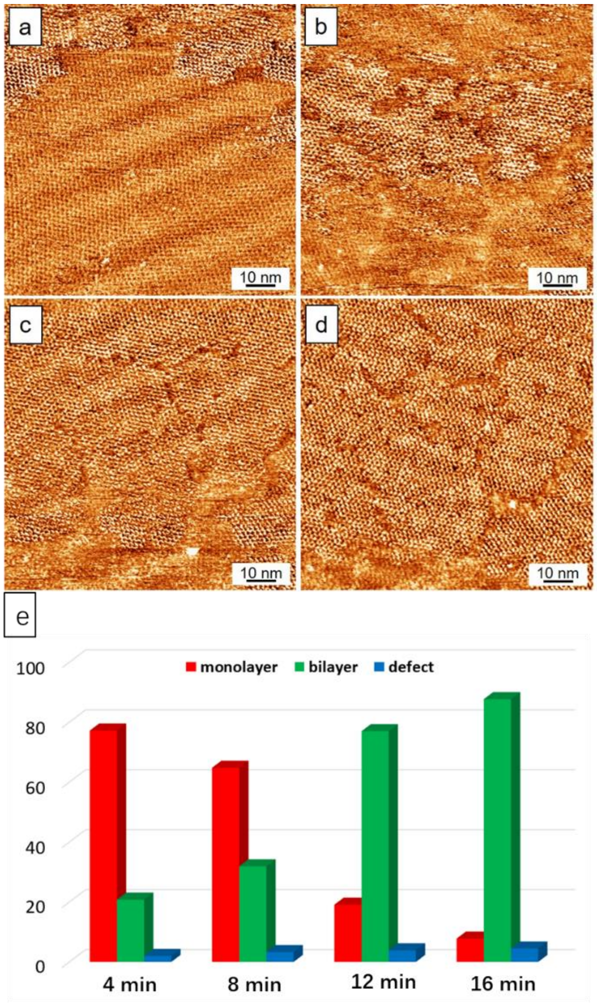

Figure S3. (a-d) Large-area STM images showing the dynamics of forming TPTC bilayers (Figure 2 in the main text). (e) Statistics of the surface coverage for the monolayers, bilayers and defects at different time. The tendency of growth for the bilayer in panel (e) is the consistent with that in Figure 2e in the manuscript.

Table S2. Surface coverage for the domains of monolayer, bilayer, and defect, corresponding to the STM images in Figure S3.

Time	Monolayer (%)	Bilayer (%)	Defect (%)
4 min	77.2	20.8	2.0
8 min	64.8	31.9	3.3
12 min	19.1	77.0	3.9
16 min	7.8	87.7	4.5

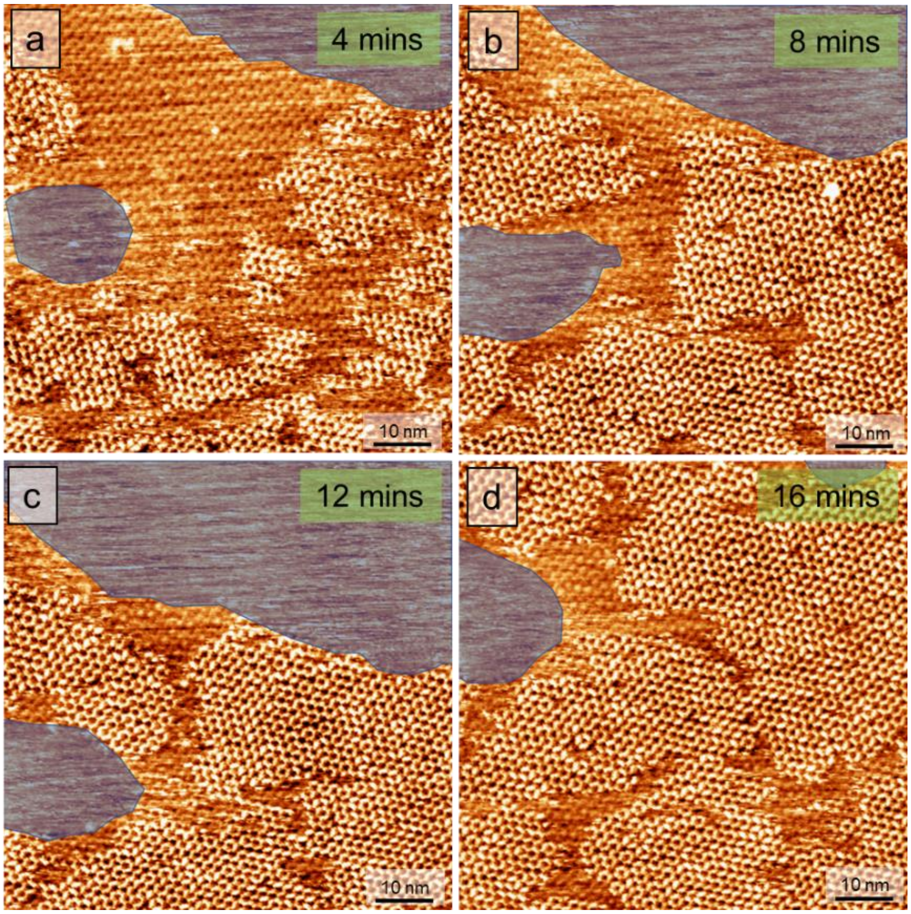

Figure S4. STM images showing the dynamics of the formation of bilayer TPTC. Scanning parameters: V_s = –0.9 V, I_t = 75 pA. The light blue area represents the defect.

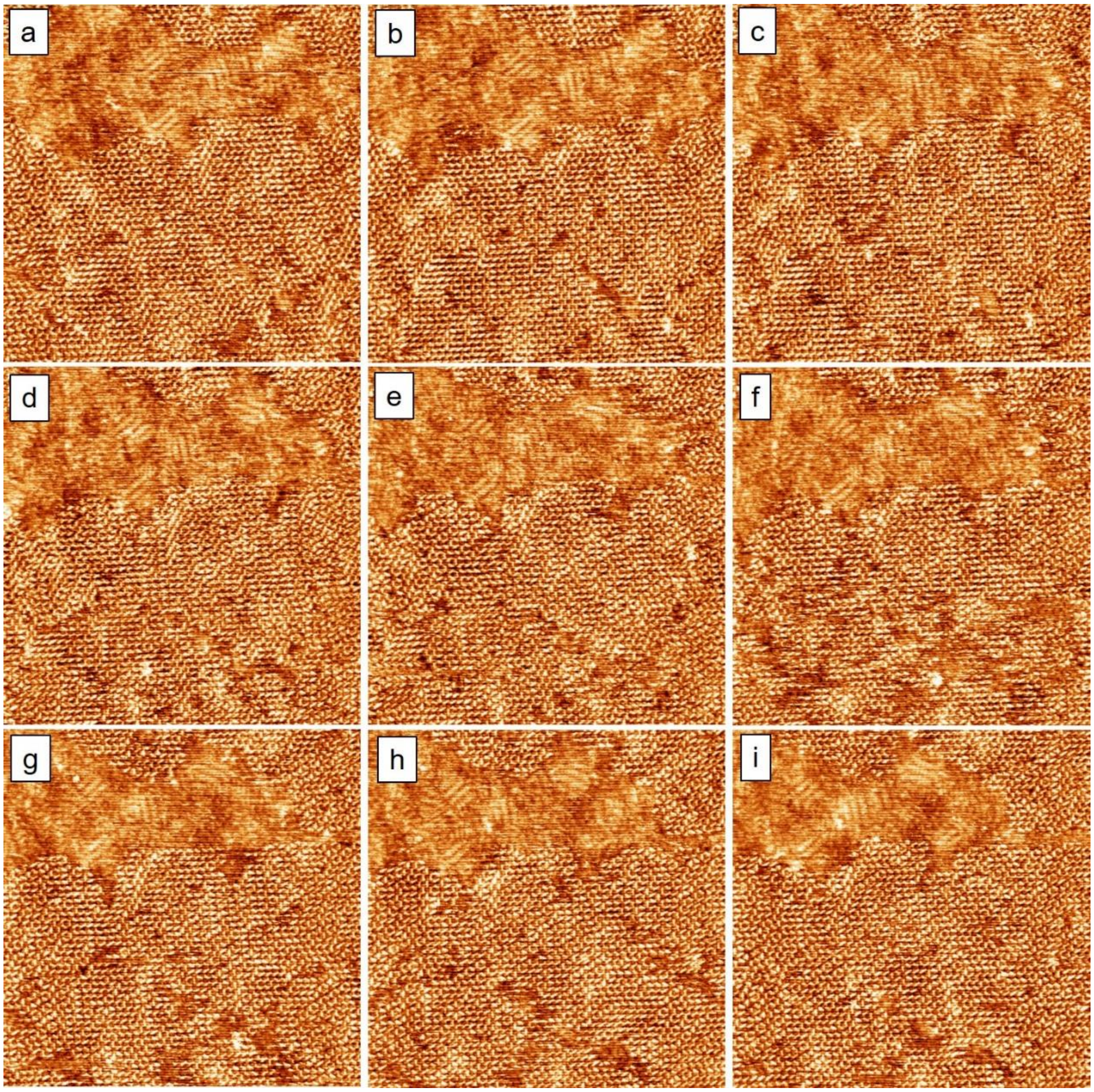

Figure S5. STM images showing the stability of bilayers. Under the positive substrate bias, that is an electric stimulation, the close packing should occur but the result shows that the bilayers remain extremely stable and not been significantly affected. Some monolayers appear on the surface ascribed to the monolayers undergo phase transition upon being subjected to the positively electric stimuli of STM. The image size is 80 × 80 nm^2. Scanning parameters: Vs = 0.9 V, It = 75 pA.

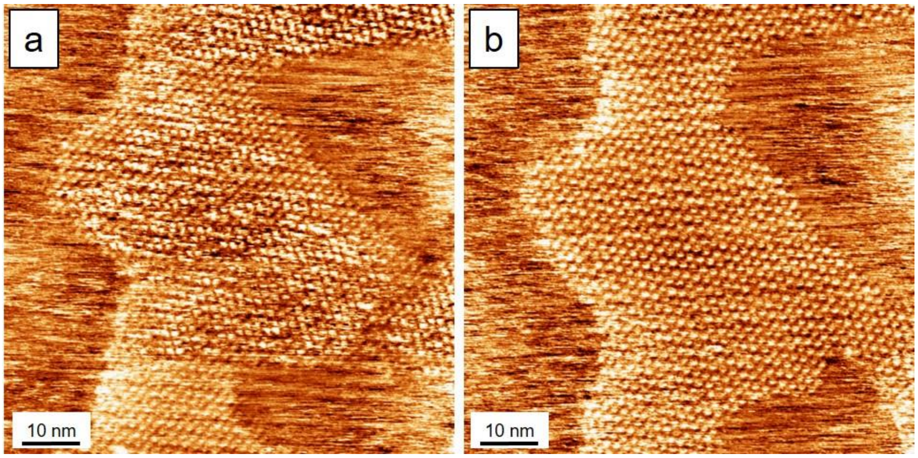

Figure S6. Reserving TPTC bilayers to monolayers via mechanical force. Here we show that the mechanical force from an STM tip can remove the upper layers, reversing the TPTC bilayers to the monolayers. The process is to increase the tunneling current from 75 to 300 pA so that the distance between the STM tip and substrate can be reduced and in turn remove the upper layers. The two images reveal the removal process. Scanning parameters: *Vs* = –0.9 V, *It* = 300 pA.

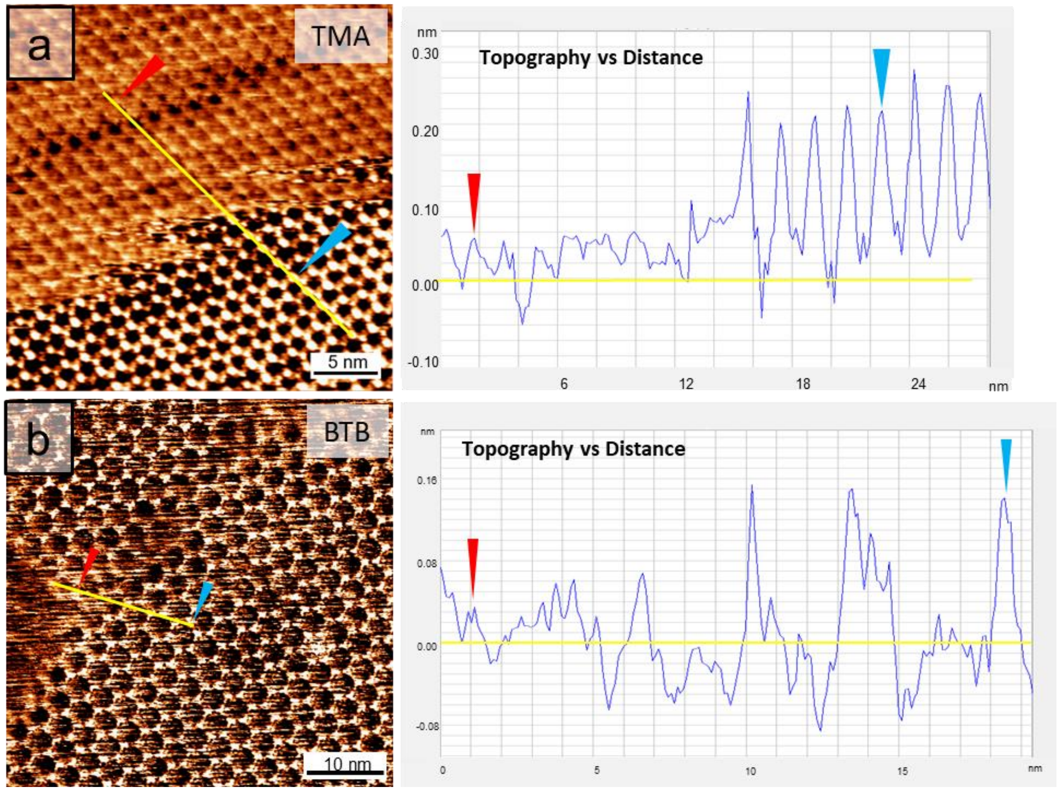

Figure S7. STM image for (a) TMA and (b) BTB and the line profiles which across the monolayer and bilayer domains.

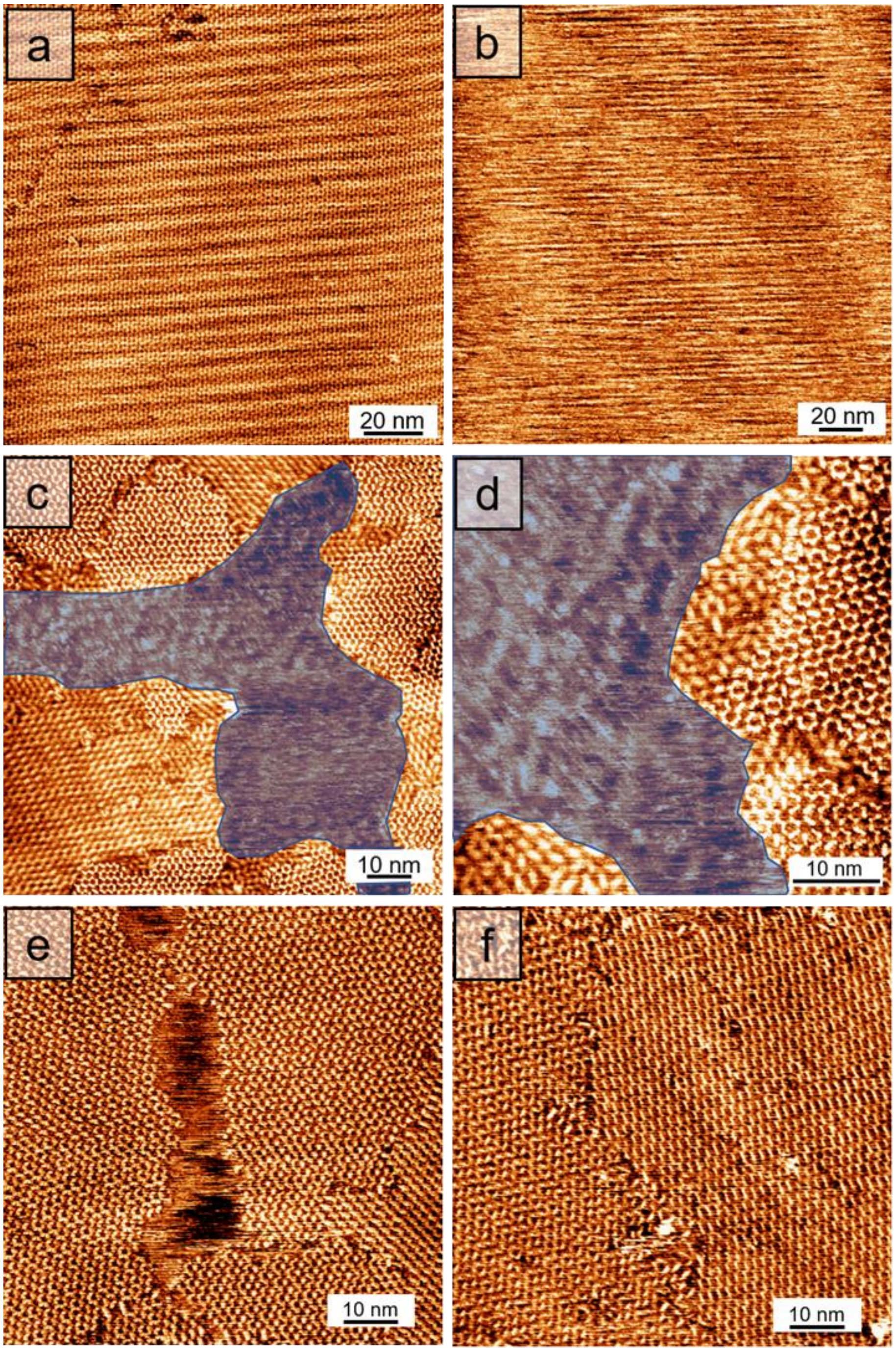

Figure S8. STM images for the self-assembly of TPTC in (a) solution I, (b) solution II, (c, d) 10% diluted solution I and solution II, (e, f) solution I and fully sonicated solution II. Scanning parameters: V_s = –0.9 V, I_t = 75 pA.

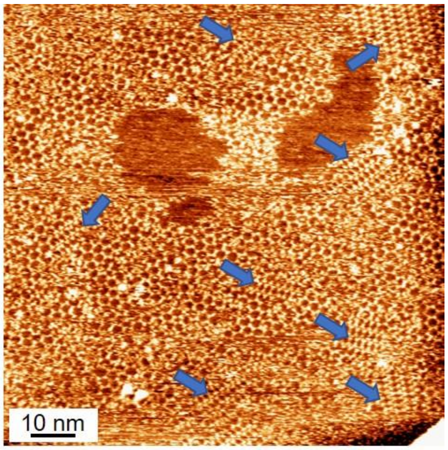

Figure S9. A surface captured 7 hours later after depositing sample I of BTB. The result can be considered as a surface where solvent has evaporated fully. STM revealed that higher density polymorphs of BTB, namely linear packing, appeared as indicated by arrows (~30%). This result suggests that the yielding of bilayers is unlikely to result from solvent evaporation although we cannot rule out its contribution during thin-film formations. V_s = –0.75 V, I_t = 100 pA.

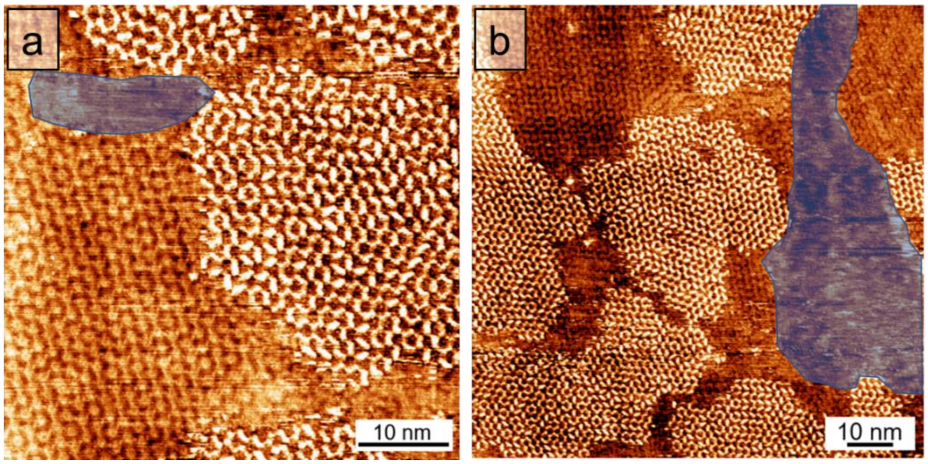

Figure S10. Solvent effect of the present system. Using 1-PO as the solvent, the result from STM investigations comes to the same conclusion with that of using OA as the solvent. Thus, the types of solvent do not cause noticeable influences. The light blue areas represent the surface without adsorbed TPTC molecules, which means the defects. Scanning parameters: *Vs* = –0.9 V, *It* = 75 pA.

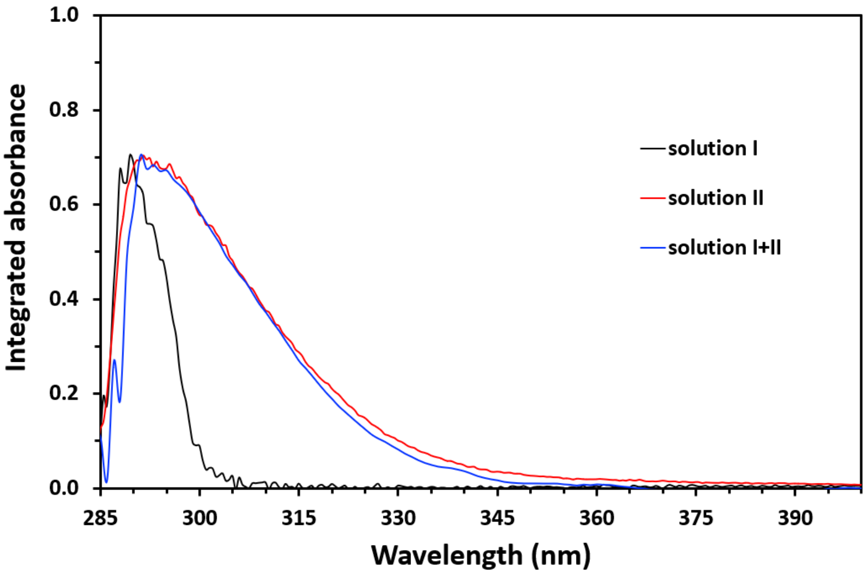

Figure S11 Spectroscopic analysis of sample solutions (normalized absorption). The UV-Vis data reveal that red-shift occurs, indicative of the aggregation behavior of the target molecules TPTC in solution II. The shift can result from molecular π-stacking and hydrogen bonding between molecules, which supports our proposal of the mechanism (less influence from solvation, Scheme 2 in the main text).
